# CSPG4.CAR-T Cells Modulate Extracellular Matrix Remodeling in DMD Cardiomyopathy

**DOI:** 10.3390/ijms26146590

**Published:** 2025-07-09

**Authors:** Maria Grazia Ceraolo, Marika Milan, Nicole Fratini, Raffaello Viganò, Salma Bousselmi, Andrea Soluri, Elisa Pesce, Pier Luigi Mauri, Giusy Ciuffreda, Elisa Landoni, Francesca Brambilla, Gianpietro Dotti, Dario Di Silvestre, Fabio Maiullari, Claudia Bearzi, Roberto Rizzi

**Affiliations:** 1 Neurology Unit, Fondazione IRCCS Ca’ Granda Ospedale Maggiore Policlinico, 20122 Milan, Italy; cmariagrazia01@gmail.com (M.G.C.); marika.88milan@gmail.com (M.M.); bousselmi@ingm.org (S.B.); 2 Department of Molecular Medicine, Sapienza University, Viale Regina Elena, 324, 00161 Rome, Italy; nicole.fratini@uniroma1.it; 3 Institute for Biomedical Technologies, National Research Council, Via Fratelli Cervi, 93, 20054 Segrate, Italy; raffaello.vigano@itb.cnr.it (R.V.); pierluigi.mauri@itb.cnr.it (P.L.M.); giusy.ciuffreda@itb.cnr.it (G.C.); francesca.brambilla@itb.cnr.it (F.B.); dario.disilvestre@itb.cnr.it (D.D.S.); fmaiullari@ichf.edu.pl (F.M.); 4 Ph.D. Program in Cellular and Molecular Biology, Department of Biology, University of Rome “Tor Vergata”, Via della Ricerca Scientifica, 1, 00133 Rome, Italy; 5 Institute of Biochemistry and Cell Biology, National Research Council of Italy (IBBC-CNR), Via Ercole Ramarini, 32, 00015 Monterotondo, Italy; asoluri10@gmail.com; 6 Fondazione Istituto Nazionale di Genetica Molecolare “Romeo ed Enrica Invernizzi”, Via Francesco Sforza, 35, 20122 Milan, Italy; pesce@ingm.org; 7 Lineberger Comprehensive Cancer Center, University of North Carolina at Chapel Hill, Chapel Hill, NC 27599, USA; elisa_landoni@med.unc.edu (E.L.); gdotti@med.unc.edu (G.D.); 8 Institute of Physical Chemistry—Polish Academy of Sciences, Marcina Kasprzaka 44/52, 01-224 Warsaw, Poland; 9 Department of Medical-Surgical Sciences and Biotechnologies, Sapienza University of Rome, Corso della Repubblica 79, 04100 Latina, Italy

**Keywords:** cardiomyopathy, immunotherapy, Duchenne muscular dystrophy, heart failure, extracellular matrix, innervation

## Abstract

Targeting fibrosis in Duchenne muscular dystrophy (DMD)-associated cardiomyopathy is a critical outstanding clinical issue, as cardiac failure remains a leading cause of death despite advances in supportive care. This study evaluates the therapeutic efficacy of CSPG4-targeted chimeric antigen receptor (CAR) T cells in reducing cardiac fibrosis and improving heart function in a preclinical model of the disease. DMD is a progressive genetic disorder characterized by degeneration of skeletal and cardiac muscle. Cardiomyopathy, driven by fibrosis and chronic inflammation, is a leading contributor to mortality in affected patients. Proteoglycans such as CSPG4, critical regulators of extracellular matrix dynamics, are markedly overexpressed in dystrophic hearts and promote pathological remodeling. Current treatments do not adequately target the fibrotic and inflammatory processes underlying cardiac dysfunction. CSPG4-specific CAR-T cells were engineered and administered to dystrophic mice. Therapeutic efficacy was assessed through histological, molecular, and echocardiographic analyses evaluating cardiac fibrosis, inflammation, innervation, and overall function. Treatment with CSPG4 CAR-T cells preserved myocardial integrity, improved cardiac performance, and reduced both fibrosis and inflammatory markers. The therapy also restored cardiac innervation, indicating a reversal of neural remodeling commonly seen in muscular dystrophy-related cardiomyopathy. CSPG4-targeted CAR-T therapy offers a novel, cell-based strategy to mitigate cardiac remodeling in dystrophic hearts. By addressing core fibrotic and inflammatory drivers of disease, this approach represents a significant advancement in the development of precision immune therapies for muscular dystrophies and cardiovascular conditions.

## 1. Introduction

Duchenne muscular dystrophy (DMD) is a severe X-linked neuromuscular disorder caused by the absence of dystrophin, leading to progressive muscle degeneration, including cardiac damage and impaired innervation [1,2]. Cardiomyopathy, a major cause of mortality in DMD patients, is characterized by excessive fibrosis driven by chronic inflammation, fibroblast proliferation, and an imbalance between extracellular matrix (ECM) production and degradation [3,4]. This process significantly contributes to heart muscle stiffness, disrupting myocardial structure and contractility while increasing the risk of arrhythmias, heart failure, and sudden cardiac death [5]. Persistent macrophage and T cell activation play a key role in ECM remodeling by releasing cytokines, chemokines, and growth factors that promote pathological collagen deposition by cardiac fibroblasts. Far from being merely structural, the ECM actively regulates inflammation and tissue remodeling, with proteoglycans and glycosaminoglycans playing crucial roles in muscle regeneration and fibrotic progression [6].

Despite growing evidence of the ECM’s involvement in DMD, no current therapies specifically target its pathological remodeling or the inflammation underlying cardiac dysfunction. Standard treatments for DMD-associated cardiomyopathy include ACE inhibitors, beta-blockers, and mineralocorticoid receptor antagonists, which reduce cardiac workload and slow disease progression [7]. Corticosteroids can also delay muscle degeneration and provide modest cardiac benefits. Emerging RNA- and cell-based therapies aim to correct the underlying genetic defect and offer potential long-term benefits [8,9].

However, these strategies do not directly address fibrosis or chronic inflammation. Notably, Givinostat, a histone deacetylase inhibitor, has demonstrated efficacy in reducing inflammation and fibrosis in both skeletal and cardiac muscle in preclinical models [10,11,12]. Following positive results from clinical trials [13], it was recently approved by the European Medicines Agency (EMA) for the treatment of DMD [14]. To date, a combined approach, including early intervention, pharmacological treatment, and advanced therapies, remains essential for managing DMD-related cardiac complications [15,16].

Recently, CAR-T cell therapy has emerged as a promising immunotherapeutic strategy for inflammatory and fibrotic diseases. Originally developed for cancer, this therapeutic approach is being repurposed for diverse pathological conditions. CAR-T cells can selectively eliminate autoreactive immune cells in autoimmune disorders such as systemic lupus erythematosus [17,18] and multiple sclerosis [19], and may also enhance immune responses to chronic infections [20,21]. Advances in CAR-T technology suggest its potential to target pathological cell populations in fibrotic conditions, including idiopathic pulmonary and cardiac fibrosis [22,23,24]. Notably, Aghajanian et al. demonstrated that T cells engineered to recognize fibroblast activation protein (FAP) on cardiac fibroblasts significantly reduced fibrosis and improved cardiac function in mice following myocardial infarction, highlighting a promising strategy to treat or even reverse cardiac disease [25,26]. In the context of DMD, chondroitin sulfate proteoglycan 4 (CSPG4) has emerged as a key pathological ECM component, accumulating abnormally in the dystrophic heart. It has also been implicated in defective sympathetic reinnervation and impaired scar reperfusion in murine models of cardiac ischemia via its interaction with protein tyrosine phosphatase sigma (PTPσ) on sympathetic axons [27]. Importantly, CSPG4 accumulation and altered sulfation patterns have been observed in muscle biopsies from DMD patients, but not in other myopathies [28]. Our previous work showed that infiltrating macrophages in dystrophic cardiac tissue overexpress CSPG4, contributing to pathological ECM remodeling and the development of dilated cardiomyopathy [11]. These findings suggest CSPG4 as a potential therapeutic target for cardiac fibrosis in DMD.

CSPG4 has already been explored as a promising CAR-T target in melanoma [29,30], glioblastoma [31,32], and leukemia [33]. Preclinical studies have shown that CSPG4-targeted CAR-T cells can effectively reduce tumor burden, demonstrating strong cytotoxicity against CSPG4-positive tumor cells even in high-antigen-density environments [34,35].

Building on this evidence, the present study investigates CSPG4-targeted CAR-T cells as a novel strategy to modulate fibrosis and improve cardiac function in DMD. We present a proof-of-concept approach demonstrating that CSPG4-directed CAR-T cells reduce fibrosis and enhance cardiac performance in a dystrophic mouse model. This strategy leverages previous research on CAR-T therapy in non-oncologic applications, including fibrosis and cardiac diseases. By targeting CSPG4, we aim to provide preclinical evidence supporting CAR-T-based interventions for DMD-associated cardiomyopathy, offering new insights into potential therapeutic approaches for DMD-related heart failure.

## 2. Results

### 2.1. CSPG4.CAR-T Generation and In Vivo Experiments

**Figure 1:**
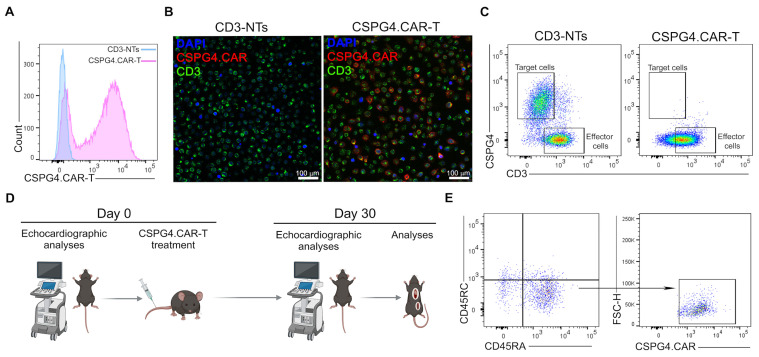
CSPG4.CAR-T generation and characterization. (**A**) Flow cytometry histogram shows CSPG4.CAR expression in CSPG4.CAR-Ts lymphocytes (pink line) compared to NTs cells (cyan line). (**B**) Immunofluorescence staining for CSPG4.CAR (red) and CD3 (green) markers. Nuclei were counterstained with DAPI (blue). Scale bars represent 100 μm. (**C**) Representative dot plots of the cytotoxic activity of CSPG4.CAR-T compared to CD3-NTs lymphocytes. Flow cytometry plots show T cells (effector on the x-axis) and U87-MG (target on the y-axis) on day 4 of co-culture. Anti-CD3 and anti-CSPG4 conjugated antibodies were used as markers for T cells and U87-MG, respectively. (**D**) Representative image of the in vivo treatment created with BioRender. (**E**) Flow cytometry analysis showing the presence of CSPG4.CAR-T lymphocytes in the spleen compared to native mouse lymphocytes, identified by CD45RA (human) and CD45RC (mouse) markers, 30 days post treatment.

CSPG4.CAR-T cells were generated from human peripheral blood mononuclear cells (PBMCs) of healthy donors, as described in the Section 4. Transduction efficiency was high, as confirmed by FACS analysis and immunofluorescence (Figure 1A,B). To assess the cytotoxic activity of CSPG4.CAR-T cells, engineered and non-transduced CD3^+^T (CD3-NTs) lymphocytes were co-cultured with U87-MG glioblastoma cells, which are characterized by high CSPG4 expression. After four days, FACS analysis demonstrated that CSPG4.CAR-T cells significantly eliminated tumor cells compared to CD3-NTs (Figure 1C). The effect of CSPG4.CAR-T on dystrophic cardiomyopathy was evaluated in vivo by administering 5 × 10^6^ T cells (either CD3-NTs or CSPG4.CAR-T) into the caudal vein of 10-month-old adult WT and *mdx* mice. To assess cardiac performance, the animals underwent echocardiography at baseline (T0) and were then randomly assigned to the following experimental groups to ensure a balanced distribution of cardiomyopathy severity: (I) WT+CD3-NTs, (II) WT+CSPG4.CAR-T, (III) *mdx*+CD3-NTs, and (IV) *mdx*+CSPG4.CAR-T. Thirty days’ post-injection, the animals were subjected to echocardiographic analysis, and the hearts were subsequently harvested for histological and molecular analyses (Figure 1D). To evaluate the persistence and biodistribution of the injected CAR-T cells, the spleen was collected at the 30-day time point. Flow cytometry analysis was performed using mouse CD45RC and human CD45RA markers to distinguish between endogenous and transferred immune cells. Among the human CD45RA^+^ cells, the expression of the CAR construct was further evaluated to confirm the presence of CSPG4.CAR-T cells in the spleen. The results demonstrated that a fraction of the injected CAR-T cells were still detectable in the spleen at sacrifice, indicating their ability to survive and potentially contribute to long-term immunomodulatory effects (Figure 1E). Additionally, the treatment was well tolerated, with no observable adverse effects.

### 2.2. CSPG4.CAR-T Cells Reduce Fibrosis and Enhance Innervation Recovery in mdx Mice

**Figure 2:**
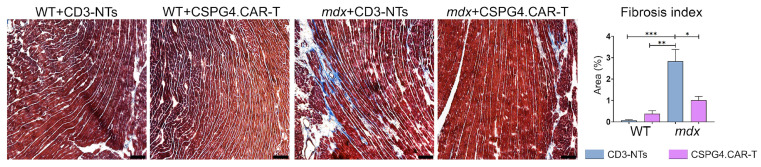
Fibrosis evaluation. Representative Masson’s trichrome images of WT and *mdx* hearts treated with CD3-NTs or CSPG4.CAR-T cells. Scale bar 100 μm. The bar chart shows the fibrotic index quantification (fibrotic area/whole area) after 30 days of CD3-NTs or CSPG4.CAR-T injection. *N* = 3 biological replicates. The repeated measure ANOVA with Tukey correction was used to evaluate the differences between means, *p*: * *p* < 0.05, ** *p* < 0.01, *** *p* < 0.001.

Histological analysis of cardiac tissue 30 days after CAR-T treatment revealed significant changes in fibrosis levels, assessed via Masson’s Trichrome staining. As previously demonstrated, *mdx* mice exhibit severe ventricular fibrosis [11], a hallmark feature that was reconfirmed in the *mdx* CD3-NTs group when compared to WT mice. Interestingly, the treatment with CSPG4.CAR-T cells was associated with a significant reduction in cardiac fibrosis (Figure 2). These findings suggest a further potential link between targeting CSPG4 and modulating fibrosis, in line with previous observations. It is also known that *mdx* mice displayed a highly disorganized distribution of sympathetic nerve terminals in the heart [11]. Moreover, our previous data indicate that the dystrophic macrophages’ (MPs) ability to secrete CSPG4 has a paracrine effect on the cardiac microenvironment, reducing synaptic terminals in a 3D in vitro model of the neurocardiac junction (NCJ). Interestingly, here we observed that beyond its effect on fibrosis, CSPG4.CAR-T treatment also influenced the sympathetic innervation. 

**Figure 3:**
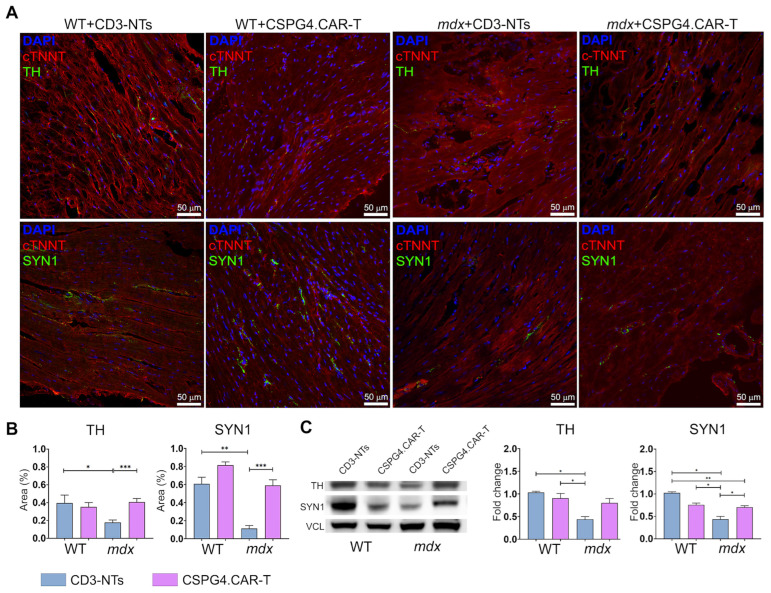
Histological analysis of cardiac innervation. (**A**) Representative confocal images of WT and *mdx* mice hearts treated with CD3-NTs or CSPG4.CAR-T cells and stained for tyrosine hydroxylase (TH) or Synapsin 1(SYN1) in green, and cardiac troponin (cTNNT) in red. Nuclei are counterstained with DAPI in blue. Scale bar 50 μm. (**B**) Charts indicate positive area expressed as percentage of TH and SYN1 on whole area. *N* = 3 section per *N* = 3 biological replicates. (**C**) Western blot analysis of TH and SYN1 in total hearts of WT and *mdx* mice. Bar charts show optical density (OD) of protein bands normalized to Vinculin (VCL). *N* = 3 biological replicates per group were analyzed. Error bars show SEM. The repeated measure ANOVA with Tukey correction was used to evaluate the differences between means, *p*: * *p* < 0.05, ** *p* < 0.01, *** *p* < 0.001.

The immunofluorescence (Figure 3A,B) and Western blot analysis (Figure 3C) suggest that CAR-T therapy partially restored cardiac innervation and nerve distribution in dystrophic mice, with a trend toward WT-like levels. In contrast, CD3-NT-treated *mdx* mice showed no comparable improvement. Importantly, the immunotherapy did not alter the expression of the innervation markers Tyrosine Hydroxylase (TH) and Synapsin 1 (SYN1) in WT mice, indicating a targeted effect only on dystrophic pathology.

**Figure 4:**
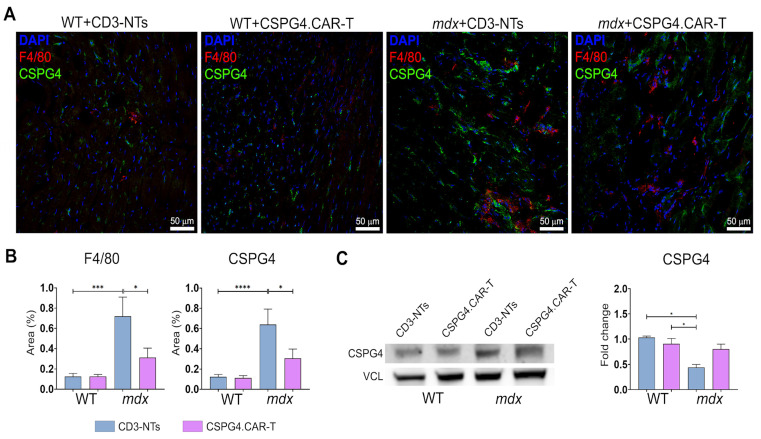
Histological analysis of cardiac inflammation. (**A**) Representative confocal images of WT and *mdx* mice’ hearts treated with CD3-NTs or CSPG4.CAR-T cells and stained for CSPG4 in green, and F4/80 in red. Nuclei are counterstained with DAPI in blue. Scale bar 50 μm. (**B**) Charts indicate positive area expressed as percentage of CSPG4 and F4/80 on whole area. *N* = 3 section per *N* = 3 biological replicates. (**C**) Western blot analysis of CSPG4 in total hearts of WT and *mdx* mice. Bar chart shows optical density (OD) of protein bands normalized to Vinculin (VCL). *N* = 3 biological replicates per group were analyzed. Error bars show SEM. The repeated measure ANOVA with Tukey correction was used to evaluate the differences between means, *p*: * *p* < 0.05, *** *p* < 0.001, **** *p* < 0.0001.

In addition to these observations, CSPG4.CAR-T therapy also led to a marked reduction in inflammation, another distinctive trait of dystrophic cardiomyopathy, as evidenced by the significant decrease in F4/80 and CSPG4-positive areas (Figure 4A,B). Moreover, Western blot analysis revealed a trend toward reduced CSPG4 expression, although the difference was not statistically significant (Figure 4C). Collectively, these results demonstrate that CSPG4.CAR-T therapy has a multifaceted therapeutic effect by reducing pathological ECM remodeling, restoring sympathetic innervation, and dampening inflammation. This triple impact highlights its potential to comprehensively counteract the structural and functional abnormalities of dystrophic cardiomyopathy.

### 2.3. CSPG4.CAR-T Cells Improve Cardiac Function in mdx Mice

**Figure 5:**
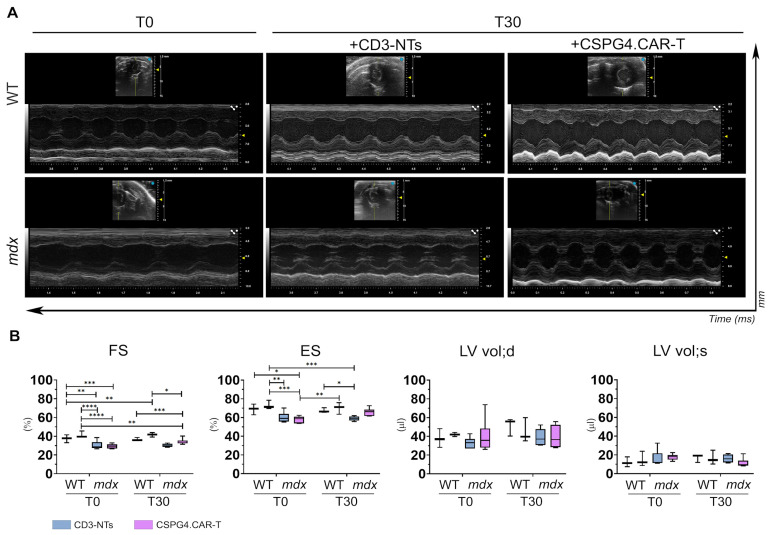
Echocardiographic analyses. (**A**) Echocardiographic M-mode images of parasternal long axis anterior and posterior walls of left ventricle of WT and *mdx* hearts treated with CD3-NTs or CSPG4.CAR-T cells, T0 left panel and T30 right panel (Vevo 3100, VisualSonics). (**B**) The graphs show the echocardiographic parameters: fraction shortening (FS), ejection fraction (EF), ventricular volume at the end of diastole (LV vol;d) and systole (LV vol;s) in the different experimental groups WT+CD3-NTs, WT+CSPG4.CAR-T, *mdx*+CD3-NTs, and *mdx*+CSPG4.CAR-T at T0 and at the end of treatment, T30. *N* = 3 biological replicates per group were analyzed. Data are represented as SEM. The *p*-values: * *p* < 0.05, ** *p* < 0.01, *** *p* < 0.001, **** *p* < 0.0001 are calculated with one-way ANOVA.

To assess the potential therapeutic benefits of CSPG4.CAR-T cells on cardiac function, echocardiographic analyses were performed at two time points: before the treatment (T0) and 30 days after the injection (T30). The graphs clearly show that the treatment with CSPG4.CAR-T cells leads to an improvement in cardiac function, as evidenced by the increase in both fractional shortening (FS) and ejection fraction (EF) in *mdx* mice treated with CAR-T cells compared to the CD3-NTs control group (Figure 5A,B). Notably, fractional shortening is slightly higher in the *mdx*+CSPG4.CAR-T group (34.95 ± 3.08) compared to the *mdx*+CD3-NTs control group (30.15 ± 1.72) at T30. While this increase is modest, it is nonetheless biologically relevant, suggesting an enhanced contractile function of the heart following CSPG4.CAR-T treatment. Similarly, the ejection fraction shows a marked improvement in the *mdx*+CSPG4.CAR-T group (65.88 ± 1.08) compared to control (58.9 ± 2.27), further supporting the beneficial effect of the therapy. Importantly, cardiac function in WT groups (WT+CD3-NTs and WT+CSPG4.CAR-T) remains stable over time. Additionally, treatment with CD3-NTs cells does not influence cardiac function in either WT or *mdx* groups, confirming that the observed benefits are specific to the engineered T cells rather than a general effect of T cell injection. Despite the moderate increase, these findings further support the therapeutic potential of CSPG4.CAR-T in preserving and improving cardiac function in the *mdx* model, highlighting its promise as a targeted intervention.

### 2.4. CSPG4.CAR-T Effects on Gene and Cytokine Profiles

**Figure 6:**
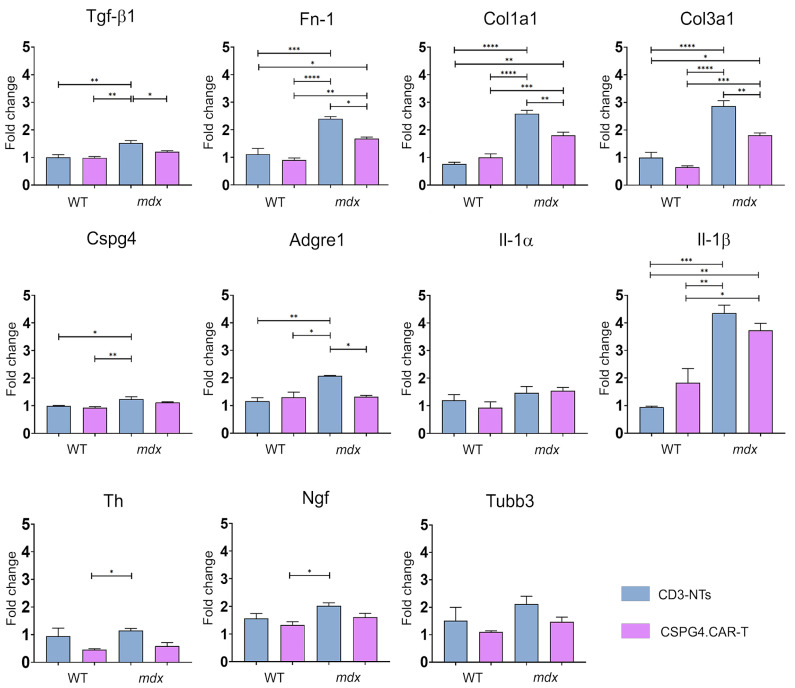
Gene expression analyses. Quantitative RT-PCR for the expression of fibrosis and ECM markers (Tgf-β1, Fn-1, Col1a1, Col3a1, Cspg4), inflammation (Adrge1, Il-1α, Il-1β), and innervation (Th, Ngf, Tubb3). *N* = 3 biological replicates per group were analyzed. Error bars represent ± SEM. The repeated measure ANOVA with Tukey correction was used to evaluate the differences between means, *p*: * *p* < 0.05, ** *p* < 0.01, *** *p* < 0.001, **** *p* < 0.0001.

To investigate the molecular effects of CSPG4.CAR-T cell therapy, we conducted a comprehensive gene expression analysis, focusing on fibrosis, inflammation, and innervation markers in *mdx* mice. Our results revealed a significant reduction in key fibrosis-related genes, including TGF-β1, fibronectin (Fn-1), collagen type 1 (Col1a1), and collagen type 3 (Col3a1), in CSPG4.CAR-T-treated *mdx* mice compared to the CD3-NTs-treated *mdx* control group, bringing their expression closer to WT levels (Figure 6). Notably, this treatment did not induce significant gene expression changes in WT mice, suggesting a disease-specific effect and reinforcing the absence of adverse consequences from engineered T cell administration in a healthy system. Beyond fibrosis, CSPG4.CAR-T therapy also influenced inflammatory markers. In CSPG4.CART-treated *mdx* mice, Adgre1 expression was significantly reduced, and Il-1β showed a slight, though not significant, decrease, suggesting that this adoptive T cell therapy may help reshape the immune landscape in DMD, particularly by modulating macrophage activity. Given that Adgre1 is a marker for macrophages, its reduction may indicate a decrease in their overall recruitment, aligning with existing evidence on immune cells’ interactions in neuromuscular disorders [36]. This is also consistent with the reduction in inflammation observed in our previous results. Interestingly, Il-1α expression remained unchanged, indicating a selective modulation of inflammatory pathways rather than a broad immunosuppressive effect. The expression of Cspg4 itself was lower in CSPG4.CAR-T-treated animals compared to controls, although not significantly. This suggests that the primary therapeutic impact might occur at the protein level rather than at the transcriptional level, as indicated by the quantification data of the immunofluorescence in Figure 4B,C. Finally, we examined the expression of innervation-related markers. Unfortunately, we did not observe significant differences in the expression levels Th, Ngf, or Tubb3 following treatment, which remained stable across all analyzed groups. Since gene expression analysis was conducted on whole heart tissue, it is possible that localized differences in neuronal remodeling were not detectable at the transcriptional level.

Alternatively, the lack of transcriptional changes observed at T30 may reflect a later time point at which gene expression has already normalized, making it less likely to capture early regulatory events. As shown in Figure 4, differences in innervation were still evident at the protein level by immunofluorescence, suggesting that earlier transcriptional alterations or post-transcriptional mechanisms may have contributed to these effects. Moreover, ECM remodeling and reduced inflammation, both significantly affected by CSPG4.CAR-T therapy, may have indirectly contributed to neuronal reorganization. Further analyses within specific cardiac regions could provide deeper insights into the regulation of innervation in this context.

**Figure 7:**
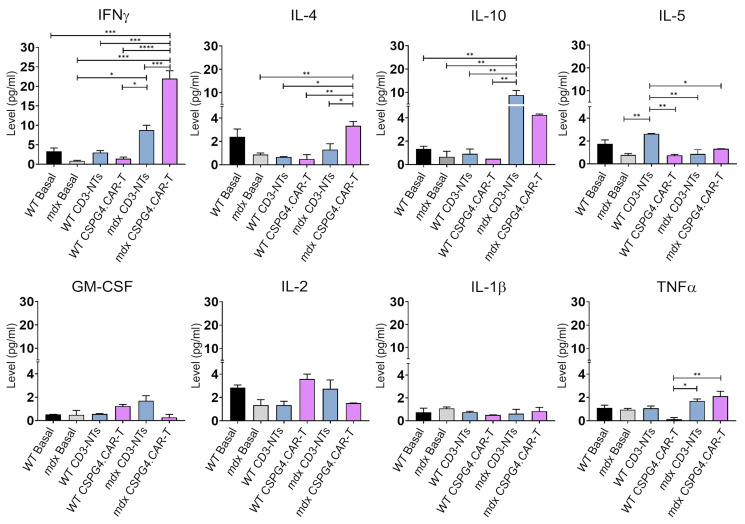
Cytokines’ profile analyses. Long-term cytokine levels after 30 days of CSPG4.CAR-T cell treatment. Basal levels were determined by the average cytokine levels of 3 untreated mice (WT black bar and *mdx* grey bar). Data are expressed as pg/mL. *N* = 3 biological replicates per group were analyzed. Error bars represent ± SEM. The repeated measure ANOVA with Tukey correction was used to evaluate the differences between means, *p*: * *p* < 0.05, ** *p* < 0.01, *** *p* < 0.001, **** *p* < 0.0001.

We also employed a multiplex immunoassay to simultaneously quantify eight key cytokines in mouse serum, after 30 days of treatment, to evaluate immune response progression and therapeutic efficacy. The baseline of cytokine levels was analyzed in untreated WT and *mdx* mice (black (WT) and gray (*mdx*) bars). Our results indicate a significant overexpression of IFN-γ in mdx mice treated with CSPG4.CAR-T cells compared to wild-type (WT) and *mdx* control groups (*mdx* basal and *mdx*+CD3-NTs) (Figure 7). This suggests an active response of engineered T lymphocytes against target cells, serving as a marker of treatment efficacy, consistent with findings in glioblastoma studies [31,32,37].

However, while this cytokine upregulation may reflect treatment efficacy, it also warrants cautious interpretation due to the known potential for IFN-γ to mediate immune-related toxicities. Additionally, we observed an increase in IL-4 levels in *mdx* treated with the CSPG4.CAR-T cells, highlighting its role in adaptive immune regulation and inflammation control. The simultaneous increase in IL-4 and IFN-γ may suggest a regulatory mechanism that balances inflammatory activity, with IL-4 promoting macrophage differentiation toward an M2-like phenotype, thereby mitigating excessive inflammation.

Notably, IL-10 levels were also elevated in the *mdx* CD3-NTs and CSPG4.CAR-T groups, suggesting its upregulation is not solely linked to CSPG4 targeting but may result from general immune activation following T cell infusion. This increase likely represents an immunoregulatory response aimed at limiting tissue damage caused by excessive inflammation. Thus, IL-10 upregulation may function as a negative feedback mechanism counteracting excessive immune activation driven by an expansion of regulatory T cells (Tregs) or M2 macrophages, both crucial for immune homeostasis. Similar findings in adoptive T cell transfer studies suggest that transient IL-10 increases help balance immune activation [38], supporting the hypothesis that its upregulation reflects an immunoregulatory adaptation rather than an adverse effect.

Conversely, no significant variations were observed in the levels of GM-CSF (Granulocyte-Macrophage Colony-Stimulating Factor), TNF-α (Tumor Necrosis Factor-alpha), IL-2 (a key cytokine in T cell proliferation and immune regulation), IL-1β (a pro-inflammatory cytokine primarily released by activated macrophages in response to cellular damage), and IL-5 (a pro-inflammatory cytokine that is responsible for the maturation, proliferation, activation, and migration of eosinophils), suggesting that these cytokines may not be necessary for the specific effects of CAR-T therapy in this scenario, or their production could be modulated to prevent side effects. Overall, the cytokine profile indicates a balanced immune response, where CAR-T cells are well tolerated and actively engaged while inflammation remains regulated to prevent immune-mediated toxicity.

### 2.5. CSPG4.CAR-T Therapy Effect on Cardiac Proteome

**Figure 8:**
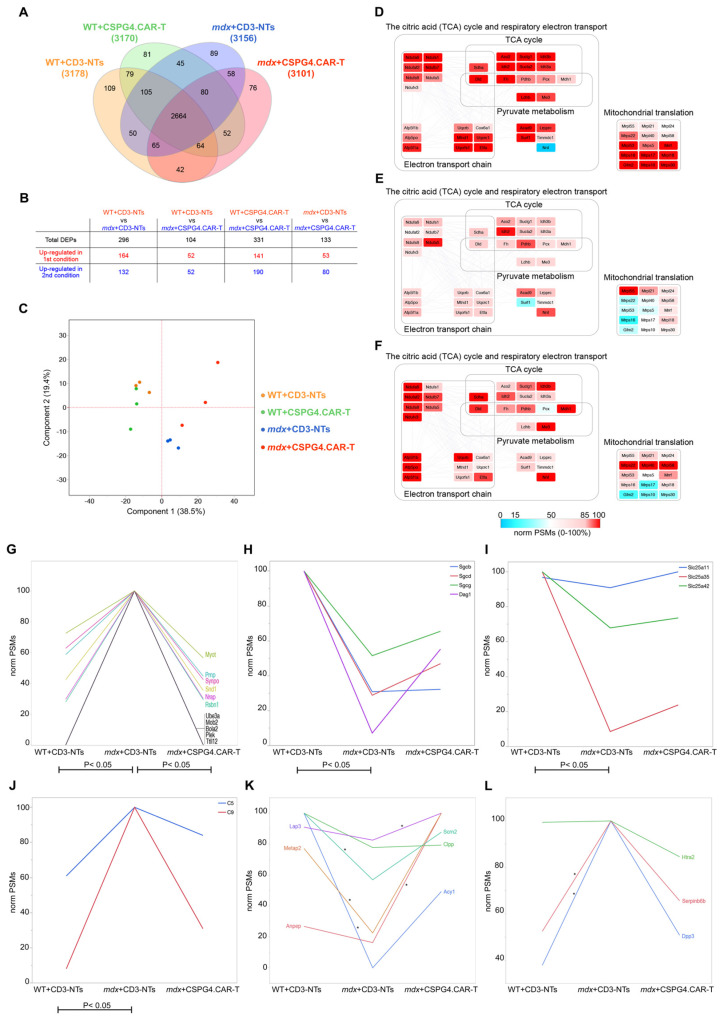
Proteomic analysis. (**A**) Venn diagram of the proteins identified in cardiac tissues from WT+CD3-NTs, WT+CSPG4.CAR-T, *mdx*+CD3-NTs, and *mdx*+CSPG4.CAR-T mice. (**B**) Differentially Expressed Proteins (DEPs) selected per pairwise comparison; DEPs were selected by means of Linear Discriminant Analysis (LDA, *p* < 0.05). (**C**) Principal Component Analysis (PCA) using DEPs selected by LDA (*p* < 0.05). (**D**–**F**) Protein–Protein Interaction (PPI) network showing DEPs belonging to TCA cycle, respiratory electron transport, and mitochondrial translation proteins in WT+CD3-NTs (**D**), *mdx*+CD3-NTs (**E**), and *mdx*+CSPG4.CAR-T (**F**). Nodes represent proteins, while links represent interaction among connected proteins; each node is colored following a color-scale grade range from light blue (0% of relative abundance) to red (100% of relative abundance). (**G**–**L**) Proteins upregulated or downregulated in *mdx*+CD3-NTs mice and recovered following CSPG4.CAR-T treatment. Selected proteins are involved in (**G**) mitochondrial proteins, (**H**) dystrophin–glycoprotein complex, (**I**) mitochondrial transport, (**J**) Complement and Coagulation Cascade, (**K**) Intracellular Peptide-Modifying Enzymes, and (**L**) Protease Modulators and Post-Transcriptional Regulators. In all panels, expression levels are visualized by norm PSMs ranging from 0% to 100% of relative abundance. *p*: * *p* < 0.05.

Following the proteomic analysis of WT and *mdx* hearts treated with either CD3-NTs or CSPG4.CAR-T cells, a total of 3.359 distinct proteins were identified (Supplementary Table S1). For each condition, the proteome comprised over 3.000 proteins, with approximately 80% shared across all groups. A small subset of proteins was uniquely detected in specific conditions (Figure 8A). The robustness of the proteomic data is underscored by the peptide-spectrum match (PSM) values for dystrophin (Dmd), which was detected in WT samples but was essentially absent in *mdx* mice, as expected (Figure S1A). The control mice, treated with CSPG4.CAR-T cells, showed minimal proteomic changes, confirming the safety and tolerability of the therapy. In contrast, *mdx* mice exhibited mild cardiac proteome remodeling following treatment (Figure 8B,C, Figure S1, Supplementary Table S2), which includes a list of statistically significant differentially expressed proteins across experimental groups.

Notably, it reactivated key metabolic pathways, particularly mitochondrial functions such as the TCA cycle, electron transport, and mitochondrial translation, which were suppressed under disease conditions and showed partial restoration after treatment (Figure 8D–F). Beyond mitochondrial pathways, CSPG4.CAR-T treatment led to the partial restoration of proteins involved in cytoskeletal integrity and synaptic regulation (Figure 8G). In particular, Pleckstrin (Plek) has been identified as a hub gene in Duchenne and Becker muscular dystrophies [39], and mutations in Myotilin (Myot) are associated with muscular dystrophy [40]. Further, proteomic analysis revealed that Myot and Nebulin-related anchoring protein (Nrap), both involved in actin cytoskeleton organization, were upregulated in *mdx* mice but decreased following treatment, suggesting a partial normalization of cytoskeletal remodeling. Proteins linked to synaptic function, including Synaptopodin (Synpo), Major prion protein (Prnp), and Ubiquitin-protein ligase E3A (Ube3a), also followed the same trend. Of particular interest, Prnp is primarily expressed in the nervous system and has been implicated in neurodegenerative diseases and mental disorders that may also affect DMD patients [41]. Synpo, on the other hand, has been proposed as a regulator of denervation-induced plasticity at hippocampal mossy fiber synapses [42]. These proteomic changes, particularly in neuro-associated proteins such as *Prnp* and *Synpo*, suggest that CSPG4-targeted therapy may influence neurocardiac signaling pathways disrupted in DMD. This is consistent with the emerging view that impaired cardiac innervation contributes to disease progression and aligns with our previous observations of CSPG4’s role in modulating neuroimmune interactions in dystrophic hearts. These observations are consistent with those from histological evaluations and gene expression analyses regarding the cardiac innervation (Figure 3). Moreover, another group of mitochondrial proteins exhibited an upregulated expression following treatment (Figure 8H,I), further supporting the idea of restored mitochondrial function. In addition, we observed a significant recovery of proteins, such as Dag1 (Dystroglycan 1), Sgcb (Sarcoglycan Beta), Sgcd (Sarcoglycan Delta), and Sgcg (Sarcoglycan Gamma), related to the dystrophin–glycoprotein complex. This suggests a compensatory mechanism that may stabilize this complex in the absence of dystrophin. Finally, the expression of two proteins from the complement and coagulation cascade (Complement C5 (C5) and Complement component C9 (C9)) was reduced following CSPG4.CAR-T treatment (Figure 8F), aligning with histological findings that indicated reduced cardiac inflammation (Figure 4A,B). Another important finding from the proteomic profiling was that CSPG4-targeted CAR-T therapy modulates key proteases and metallopeptidases involved in proteostasis and ECM remodeling (Figure 8K), restoring their expression to wild-type levels. Of note, Clpp (Caseinolytic Mitochondrial Matrix Peptidase Proteolytic Subunit), a mitochondrial protease involved in protein quality control, regulates mitochondrial proteostasis relevant to cardiomyopathy, and its downregulation has been demonstrated to mitigate cardiomyopathic features in preclinical models [43]. Dpp3 (Dipeptidyl Peptidase 3) (Figure 8L), a dipeptidyl peptidase associated with oxidative damage and cardiovascular dysfunction, was found to be overexpressed in dystrophic hearts but returned to near-normal levels following CSPG4.CAR-T treatment. Given that elevated circulating Dpp3 is a known biomarker of adverse outcomes in heart failure, its reduction may reflect decreased cardiac stress and improved tissue homeostasis [44,45]. The normalization of *Dpp3* levels following CAR-T treatment not only supports reduced cardiac stress but also indicates that CSPG4 inhibition may indirectly restore proteostasis mechanisms associated with oxidative damage in dystrophic myocardium, further reinforcing its therapeutic relevance. Finally, the modulation of multiple peptidases in *mdx* hearts suggests an adaptive response aimed at enhancing proteolytic clearance and tissue remodeling. This rebalancing of proteolytic and stress-related pathways may contribute to extracellular matrix stabilization and support antifibrotic mechanisms. Overall, the proteomic data highlight a broader remodeling effect of CSPG4.CAR-T therapy, extending beyond direct fibrotic targets and aligning with previous histological and molecular evidence. These findings further support its therapeutic potential in improving the cardiac phenotype in *mdx* mice.

## 3. Discussion

This study shows that CSPG4-targeted CAR-T cell therapy attenuates cardiac fibrosis and improves cardiac function in DMD-associated cardiomyopathy. Since excessive ECM deposition drives myocardial stiffness and dysfunction in DMD hearts, CSPG4.CAR-T treatment was found to markedly reduce the fibrotic area based on histology and downregulate key profibrotic genes (Tgf-β1, Fn1, Col1a1, Col3a1), indicating effective ECM remodeling. While our data primarily highlight a reduction in matrix synthesis pathways, it is important to note that extracellular matrix remodeling also involves active degradation, often mediated by matrix metalloproteinases (MMPs) such as MMP-2 and MMP-14. Although this study does not directly investigate the degradative axis, previous work in melanoma models has shown that CSPG4 can facilitate cell invasion through activation of membrane-bound MMP complexes, potentially including MMP-14, at the cell surface [46]. Whether a similar mechanism contributes to matrix remodeling in dystrophic cardiac tissue remains to be elucidated. Future studies will be needed to determine whether CSPG4.CAR-T therapy also modulates ECM degradation pathways in the dystrophic heart. Beyond antifibrotic effects, CSPG4.CAR-T therapy also promoted the recovery of cardiac innervation. DMD hearts exhibit disrupted sympathetic networks that exacerbate disease progression and autonomic dysfunction [11]. Here, we showed that post-treatment nerve-terminal distribution was restored to a pattern comparable with wild-type controls, and TH expression, a marker of sympathetic fibers, was significantly increased, further supporting the therapeutic impact of CSPG4.CAR-T cells in neuroimmune modulation. These findings suggest that ECM remodeling by CAR-T cells may indirectly enable restoration of tissue architecture that supports neurovascular integration, an underexplored mechanism in dystrophic cardiomyopathy. Moreover, it is known that CSPG4 accumulates in the *mdx* heart and co-localizes with inflammatory infiltrates [11], exerting an inhibitory effect on innervation. This is in line with recent clinical evidence showing that chronic inflammation persists in DMD patients despite standard care, potentially contributing to an altered tissue environment that hampers proper neurovascular interactions [47]. Such inflammatory dysregulation may further exacerbate CSPG4-mediated inhibition of cardiac innervation. Its targeted depletion via CAR-T therapy facilitates both nerve restoration and a reduction in chronic inflammation. By addressing these interconnected pathological features, CSPG4.CAR-T emerges as a promising strategy for restoring cardiac integrity in *mdx* mice and, potentially, in patients with dystrophic heart disease. The treatment also improved FS and EF in *mdx* mice, confirming enhanced cardiac performance. The association between reduced fibrosis, restored innervation, and improved contractility suggests a multifaceted mechanism, where structural, electrophysiological, and mechanical changes synergize to improve cardiac performance.

Additionally, both molecular and serum analyses revealed an immunomodulatory effect following CSPG4.CAR-T cell infusion. Specifically, the expression of macrophage-related genes such as Adgre1 and Il1b was reduced, while serum levels of IFN-γ, IL-4, and IL-10 increased. This profile indicates a balanced immune environment that supports fibrosis resolution without evidence of excessive pro-inflammatory activation. Importantly, no significant changes were observed in classic pro-inflammatory cytokines such as TNF-α, IL-2, and IL-1β, suggesting that CSPG4.CAR-T treatment does not elicit broad systemic inflammation. Although IFN-γ elevation indicates active CAR-T function, sustained high levels could pose risks like cytokine release syndrome. In this study, the absence of clinical toxicity and stable levels of other cytokines suggest a balanced immune response, highlighting the need for cytokine monitoring in future work. These findings, including the persistence of CAR-T cells in the spleen up to 30 days’ post-infusion and the absence of observable side effects, even in the context of human CAR-T cells infused into immunocompetent mice, support the notion that the therapy is well tolerated and may contribute to reprogramming the myocardial immune niche towards a reparative phenotype.

Finally, proteomic profiling showed treatment-induced shifts in protein expression. Although core ECM proteins remained largely unchanged, key pathways related to mitochondrial function, energy metabolism, and cytoskeletal organization were partially restored, alongside the modulation of proteolytic enzymes involved in peptide processing and protein turnover. These changes are consistent with histological and transcriptomic findings, indicating that CSPG4.CAR-T cells contribute to functional cardiac remodeling in dystrophic hearts. Importantly, post-treatment upregulation of cardiac energy-metabolism pathways aligns with the functional gains observed by echocardiography. These findings suggest that CSPG4.CAR-T therapy activates proteostatic mechanisms that contribute to the rebalancing of cellular homeostasis, potentially supporting improved cardiac resilience in dystrophic conditions. We hypothesize that CSPG4.CAR-T therapy may act by halting the fibrotic process through targeted disruption of pathological CSPG4 deposition while simultaneously promoting the restoration of cellular and metabolic homeostasis. In a chronically diseased and aged heart, such stabilization of the microenvironment may be sufficient to support functional recovery and enhance tissue resilience, even in the absence of major structural reversal.

Given the complexity of cardiac fibrosis in DMD, various antifibrotic strategies are currently being explored to mitigate disease progression. For example, Givinostat, a histone deacetylase inhibitor, has been recently approved by the European Medicines Agency (EMA) for Duchenne muscular dystrophy and has shown promise by modulating gene expression patterns involved in fibrosis and inflammation. Monoclonal antibodies targeting connective tissue growth factor (CTGF) represent another approach, aiming to directly inhibit key profibrotic signaling pathways. In contrast, our CSPG4-targeted CAR-T cell therapy offers a distinct and multifaceted approach. By specifically depleting CSPG4-expressing pathological ECM components, the therapy not only disrupts ongoing fibrotic matrix deposition but also fosters restoration of cardiac innervation and rebalances the immune microenvironment. This dual action on structural remodeling and immune modulation differentiates CSPG4.CAR-T cells from current pharmacological agents, potentially providing synergistic benefits when used alone or in combination with other antifibrotic treatments.

This comparative perspective highlights the innovative nature of our approach and its potential to complement existing therapies, ultimately contributing to a more effective management of dystrophic cardiomyopathy. Despite these promising results, several challenges must be addressed before clinical translation. The long-term safety, potential off-target effects, in vivo persistence of CAR-T cells, and immune-related toxicities remain to be evaluated in human patients. Moreover, while CSPG4 is validated here as a critical ECM target, complementary targets may further enhance therapeutic efficacy. We also acknowledge that the relatively small sample size (*N* = 3 per group) used in the in vivo experiments, although consistent with common practice in preclinical CAR-T research, may limit the statistical power and contribute to biological variability. Future studies with larger cohorts and additional statistical validation methods will be important to strengthen the robustness and reproducibility of these findings.

## 4. Materials and Methods

### 4.1. Generation of CAR-T Cells from Human PBMC Characterization

The CAR.CSPG4 plasmid, provided by Prof. Dotti, was amplified and used for retroviral production and infection, as previously described [48,49]. Briefly, transient retroviral supernatant was generated by transfection of the Phoenix-AMPHO cells line (ATCC, CRL-3213™, Manassas, VA, USA) with the retroviral vector using CalPhos™ Mammalian Transfection Kit (Takara Biochemicals, 631312, Kusatsu, Japan). For the generation of CAR-T cells, peripheral blood mononuclear cells (PBMCs) were isolated from human buffy coat preparations using Ficoll-Paque (Cytiva, GE17-5446-52, Buccinasco, Milan, Italy) and T-lymphocytes were enriched by using the human Pan T Cell Isolation Kit (Miltenyi, 130-096-535, Bergisch Gladbach, Germany). Total T-lymphocytes were activated with Dynabeads™ Human T-Activator CD3/CD28 (Gibco, 11131D, Waltham, MA, USA) for 48 h, following the manufacturer’s protocol. PBMCs were grown in a complete growth medium, composed of RPMI 1640 w/L-Glutamine (L0500, BioWest, Nuaillé, France) supplemented with 10% Fetal Bovine Serum (FBS, 10270106, Gibco, Waltham, MA, USA), 1% penicillin/streptomycin (P/S stock solution 10,000 U/mL, 15140122, Gibco), 1% non-essential amino-acid (NEAA, 100x solution, 11140050, Gibco), 1% Sodium Pyruvate (100 mM, 11360070, Gibco), 30 U/mL of human IL-2 (Miltenyi, 130-097-745, Bergisch Gladbach, Germany) at 37 °C, and 5% CO_2_. Non-tissue-coated 24-well plates were coated with 1 mg/mL retronectin (Takara Biochemicals, T100A, Shiga, Japan) overnight. The wells were coated with retroviral supernatant, following the Takara protocol. Subsequently, stimulated T cells were added. Cells were incubated for 2 days on the virus-coated plate in the presence of 100 U/mL IL-2, 10ng/mL of IL-7 (Miltenyi, 130-095-362), and 4 ng/mL of IL15 (Miltenyi, 130-095-764). Then T cells were transferred to a tissue-culture-treated plate and then expanded in complete medium containing IL-2, IL-7, and IL-15.

### 4.2. CAR-T Cells’ Characterization

Transduction efficiency was evaluated by FACS analysis. Cells were labeled with a CAR-specific antibody (Jackson ImmunoResearch, 115-606-072, Baltimore, MD, USA) for 30 min at RT in the dark, washed with FACS buffer, and acquired using BD FACSCanto™ II (BD Bioscience, San Jose, CA, USA). The analysis was carried out with Flowjo software version 10.9 (BD Biosciences).

Transduced T-lymphocytes were characterized also by an immunofluorescence assay against the CAR-specific antibody (Jackson ImmunoResearch, 115-606-072, Baltimore, MD, USA). Cells were collected and labeled with anti-CD3 (1:300, BioLegend, 300406, San Diego, CA, USA) for 30 min at 37 °C. Then, lymphocytes were seeded onto an adhesion microscope slide (Epredia, J1800AMNZ, Gerhard Menzel GmbH, Braunschweig, Germany) for 15 min at 37 °C and subsequently fixed in 4% Paraformaldehyde (PFA, 047347.9M1, Thermo Scientific, Waltham, MA, USA) for 15 min at room temperature and washed three times with PBS. Finally, they were stained with the CAR-specific antibody for 30 min at 37 °C and with Hoechst 33342 (1:1000, H1399, Invitrogen, Carlsbad, CA, USA) for an additional 20 min. Images were acquired using a confocal microscope TCS SP5 (Leica Microsystems, Wetzlar, Germany) and analyzed using ImageJ software version 1.53t [Rasband, W.S. (1997–2015) ImageJ, National Institutes of Health, Bethesda, MD, USA, https://imagej.net/ij, (accessed on 10 June 2020)].

The cytotoxic activity of CSPG4.CAR-transduced lymphocytes and non-transduced CD3^+^ lymphocytes (CD3-NTs) was assessed in vitro by co-culturing them with the CSPG4-expressing glioblastoma cell line U87-MG (ATCC, HTB-14™, Manassas, VA, USA) for four days at an Effector–Target (E:T) ratio of 1:1. After incubation, cells were collected and stained with anti-CD3 (1:100, Miltenyi, 130-114-519, Bergisch Gladbach, Germany) and anti-CSPG4 (1:100, Miltenyi, 130-116-376, Bergisch Gladbach, Germany) conjugated antibodies to distinguish T cells and tumor cells, respectively. Immune-mediated cytotoxicity was analyzed by flow cytometry (BD FACSCanto™ II, Becton, Dickinson and Company, San Jose, CA, USA), and killing activity was quantified based on the loss of CSPG4 fluorescence using FlowJo version 10.9 software.

### 4.3. Echocardiogram

Mice were anesthetized with isoflurane (1.2–2.0% in 100% oxygen) and maintained at 37 °C. Imaging was performed on days 0 and 30 using VisualSonics Vevo 3100 (Fujifilm, Amsterdam, The Netherlands), as previously described [50].

### 4.4. Mice Treatment

Ten-month-old C57BL/10 wild-type (WT) and C57BL/10 dystrophic mice (*mdx*) were obtained from Charles River Laboratories (Wilmington, MA, USA). All procedures were performed in conformity with the experimental protocol approved by the Ministry of Health (Protocol 1188/2020). Mice were handled in compliance with the European Convention on Animal Care (Directive 2010/63/EU of the European Parliament on the protection of animals used for scientific purposes). A total of 5 × 10^6^ CSPG4.CAR or CD3-NTs lymphocytes were resuspended in sterile phosphate buffer saline (PBS, L0615-500, BioWest, Nuaillé, France) and intravenously infused into mice via the caudal vein. Thirty days after therapy and after echocardiographic analysis, the animals were sacrificed.

### 4.5. Spleen Processing

The animals were anesthetized with tiletamine/zolazepam and xylazine. The spleen was harvested from mice 30 days post-treatment and immediately frozen. The tissue was then processed into a single-cell suspension for counting and antibody staining. Briefly, the spleen was rapidly thawed in a water bath, placed on a 70 μm filter set on a 50 mL conical tube, and mechanically dissociated using a syringe plunger. The filter was washed twice with RPMI + 2% FBS, and the sample was centrifuged at room temperature (RT) for 5 min at 500g. The resulting single-cell suspension was resuspended in cold FACS buffer, counted, and stained with the following antibodies: anti-CD45RC (1:100, BD Biosciences, 746515, San Jose, CA, USA), anti-CD45RA (1:100, BioLegend, 304128, San Diego, CA, USA), and a CAR-specific antibody. Staining was performed for 30 min at RT, after which cells were fixed with 2% paraformaldehyde (PFA), acquired using a BD FACSCanto™ II, and analyzed with FlowJo version 10.9 software.

### 4.6. Histological Analysis

Hearts were explanted 30 days post-treatment. For this purpose, the animals were anesthetized and perfused with 50 mM KCl (Sigma, P9333, St. Louis, MO, USA) via abdominal aortic cannulation. Samples were embedded in Tissue-Tek O.C.T. (VWR International S.r.l., 361603E, Milan, Italy), frozen in pre-cooled isopentane (Sigma, 277258, St. Louis, MO, USA), and sectioned into pieces with 8 μm thickness using a cryostat (Leica CM1850, Wetzlar, Germany). Sections were blocked for 30 min at RT with 5% bovine serum albumin (BSA, A1470, Sigma-Aldrich, Saint Louis, MO, USA) and incubated overnight at 4 °C with the following primary antibodies diluted in 0.5% BSA solution: anti-cardiac troponin T (cTNNT, 1:100, Abcam, ab33589, Cambridge, UK), anti-tyrosine hydroxylase (TH, 1:100, Sigma-Aldrich, ab152, St. Louis, MO, USA), anti-Synapsin 1 (SYN1, 1:100, CST, #6710, Danvers, MA, USA), anti-MPs F4/80 (F4/80, 1:100, Biorad, MCA497G, Segrate, Milan, Italy), and anti-CSPG4 (1:200, Novus Biologicals, NBP266979, Centennial, CO, USA). Then, sections were washed twice with PBS for 5 min and incubated with appropriate fluorescent-conjugated secondary antibodies (1:1000, Invitrogen, Carlsbad, CA, USA) for 1 h. Cell nuclei were counterstained with DAPI (4′, 6-Diamidino-2-Phenylindole, D1306, Life Technology, Invitrogen, ThermoFisher Scientific, Waltham, MA, USA), and the slides were mounted with ProLong Glass Antifade Mountant (P36984, Invitrogen, Carlsbad, CA, USA). Images were acquired using a confocal microscope TCS SP5 (Leica Microsystems, Wetzlar, Germany). The fibrotic index was determined using Masson’s Trichrome assay (AUS240, Bioptica, Milan, Italy) staining, following the manufacturer’s protocol. Scar size was expressed as the ratio of the fibrotic area to the total area. All images were analyzed using ImageJ [Rasband, W.S. (1997–2015) ImageJ, National Institutes of Health, Bethesda, MD, USA, https://imagej.net/ij, (accessed on 10 June 2020)].

### 4.7. Western Blot

Proteins were extracted using Lysis buffer [50 mM Tris HCl (pH 7.5), 150 mM NaCl, 5 mM EDTA, 1% NP-40, 0,1% SDS] supplemented with cOmplete™ ULTRA, Mini, EASYpack (05892970001, Roche, Basel, Switzerland). Mice’ hearts were resuspended in lysis buffer and homogenized using TissueRuptor II (120 V, 60 Hz, 9002755, QIAGEN, Germantown, MD, USA). Samples were incubated on ice for 1 h and centrifugated at 10,000× *g* for 10 min. Proteins were quantified using Qubit Protein Assay Kit on a QubitTM 4 Fluorometer (Invitrogen, ThermoFisher Scientific, Waltham, MA, USA) and 30 µg was resolved by SDS-PAGE and transferred to nitrocellulose membranes (Catalogue No. GE10600079, Amersham Protran, Johns Creek, GA, USA). Membranes were blocked in 5% non-fat milk (Catalogue No. 1.15363, Merck Millipore, Billerica, MA, USA) and incubated overnight at 4 °C with the appropriate primary antibody. Membranes were probed with the following primary antibodies: tyrosine hydroxylase (1:1000, ab152), Synapsin 1 (1:1000, #5297, Cell Signaling Technology, Danvers, MA, USA), CSPG4 (1:1000, #52635, Cell Signaling Technology, Danvers, MA, USA), and Vinculin (1:500, V9131, Sigma-Aldrich), followed by incubation with secondary antibodies (antiRabbit NA934AV, 1:5000; anti-Mouse LNXa931/AE, 1:10,000, GE Healthcare Life Sciences, Havelock, NE, USA) for 1 h at RT. Immunocomplexes were detected by chemiluminescence (ECL kit; Merck Millipore) using the ChemiDoc™ MP Imaging System (Bio-Rad Laboratories, 12003154, Hercules, CA, USA). Analyses were performed by using Bio-Rad Image Lab 5.2.1 Software.

### 4.8. Gene Expression Analysis

Total RNA was extracted from hearts using miRNeasy Plus Universal Mini Kit (Qiagen, 217004, Hilden, Germany) according to the manufacturer’s protocol. RNA concentration was determined using a NanoDrop 2000 UV–visible spectrophotometer (Thermo Scientific™, ND-2000, Waltham, MA, USA). Samples were treated with DNase I (Invitrogen, 2358609, Carlsbad, CA, USA) following the protocol and 1 μg of total RNA was reverse transcribed into cDNA using a TaqMan Reverse Transcription Reagents kit (Applied Biosystems, N8080234, Foster City, CA, USA). Gene expression was evaluated by quantitative real-time PCR (RT-qPCR) with SYBR green (SensiFAST SYBR Lo-ROX kit, Meridian Bioscience, BIO94005, Cincinnati, OH, USA) on a Real-Time PCR Instrument (Applied Biosystems Quantstudio 5 real-time PCR system, A28135/A28140, Foster City, CA, USA). The thermal cycling conditions for all primers were as follows: 2 min at 50 °C, 10 min at 95 °C, and 40 cycles of 15 s at 95 °C and 1 min at 60 °C. Data are presented as relative expression normalized to the reference transcript Eef1a2 and the WT + CD3-NTs group as control. The primer sequences are listed in Table 1.

### 4.9. Cytokine Assay

Blood was drawn with a syringe from the tail at day 30 post treatment and spun down to isolate serum. Cytokine levels in serum samples were determined using the Bio-Plex Pro Mouse Cytokine 8-plex Assay (Bio-Rad Laboratories, M60000007A, Hercules, CA, USA), a multiplex bead-based immunoassay that allows for the simultaneous quantification of eight different cytokines (GM-CSF, IFN-γ, IL-1β, IL-2, IL-4, IL-5, IL-10, TNF-α). The assay was conducted following the manufacturer’s protocol. Briefly, serum samples were diluted in the appropriate buffer and incubated with a mixture of magnetic beads, each coated with a specific capture antibody corresponding to one of the cytokines in the panel. After incubation, the beads were washed and a detection antibody, conjugated with a biotinylated secondary antibody, was added. The complexes were incubated and then detected using streptavidin–phycoerythrin (SAPE), which binds to the biotin and generates fluorescence. The fluorescence intensity was measured using the Bio-Plex 200 System (Bio-Rad Laboratories, Reader Serial Number: LX10006124403). Cytokine concentrations were determined by comparing sample fluorescence data to standard curves generated using known concentrations of recombinant cytokines provided by the assay kit. Results were expressed as pg/mL for each cytokine.

### 4.10. Proteomic Analysis

Mouse Heart Samples’ Preparation: Mouse hearts were thawed and weighed into conical bottom glass vials; 2.5 µL of Universal Nuclease (ThermoFisher Scientific, Waltham, MA, USA) and 1 mL of Lysis Solution (ThermoFisher Scientific, Waltham, MA, USA) were added to each sample. Hearth samples were all mechanically homogenized in order to disrupt tissue; homogenized tissues were then centrifuged at 13,000 rpm for 10 min. Each supernatant was recovered, and the protein content was evaluated by using the Qubit protein assay performed with Qubit4 fluorimeter (Invitrogen, part of ThermoFisher Scientific, Waltham, MA, USA). In total, 100 µg of proteins for each sample were reduced, alkylated, digested with Tryp/LysC enzymes, and, finally, cleaned up according to the protocol of the EasyPep Mini MS sample preparation kit (ThermoFisher Scientific, Waltham, MA, USA). Each digested sample was dried after a clean-up procedure and reconstituted in 0.1% formic acid (Sigma-Aldrich Inc., St. Louis, MO, USA) at a concentration of 1 µg/µL; samples were then diluted at 0.1 µg/µL before the nLC-hrMS/MS analyses.

nLC-hrMS/MS Analysis: Digested samples were separated using the Vanquish Neo UHPLC System (ThermoFisher Scientific, San Jose, CA, USA); this chromatographical system was implemented with PepMap Neo trap column (300 μm × 5 mm, Thermo Scientific, Waltham, MA, USA) and EASY-Spray™ PepMap™ Neo Column (75 μm I.D, 5 μm, 15 cm; ThermoFisher Scientific, San Jose, CA, USA) for peptide separation, working in trap-and-elute mode (Flush Direction of trap column: forward). The Vanquish autosampler parameters were: temperature set at 7 °C, loading volume of 4 µL; loading flow rate of 50 µL/min. For HPLC, the following solvents were used: eluent A, H2O with 0.1% FA, and eluent B, 80/20 ACN/H_2_O (%, *v*/*v*) with 0.1% FA. A 90-min gradient (excluding ca. 1.4 min of sample pickup and sample loading) was used for the separation phase set as follows: 4% to 5% B in 1 min, 5% to 29% B in 54 min, 29% to 50% B in 34 min, and 50 to 70% B in 1 min. The column wash phase was from 90 to 96 min at 99% B. The flow rate during the gradient was set at 300 nL/min. Mass spectra of heart samples were acquired in two technical replicates using a ThermoFisher Scientific Orbitrap Exploris™ 240 Mass Spectrometer for high-throughput bottom-up proteomic profiling. The Orbitrap Exploris 240 mass spectrometer was equipped with the NSI Easy Spray source (Thermo Fisher Scientific, San Jose, CA, USA) working in a positive ion mode (positive ion spray voltage static at 1900 V, ion transfer tube temperature 280 °C, 35 °C). The Orbitrap Exploris™ 240 Mass Spectrometer acquired MS spectra in Data-Dependent Acquisition (DDA) mode (custom, top 20 scans) according to the parameters reported in Table 2.

*Data Analysis*: Mass spectra obtained from the mass spectrometry experiments were analyzed using Proteome Discoverer 2.5 software (Thermo Fisher Scientific, San Jose, CA, USA) for protein identification. Briefly, a Processing Workflow was built with the following nodes: (1) Spectrum Files RC; (2) Spectrum Selector; (3) Minora Feature Detector; (4) Precursor Detector; (5) Spectrum Properties Filter; (6) Sequest HT as search engine; (7) INFERYS Rescoring; and (8) Percolator. The Mus musculus UniProt database retrieved in March 2025 was used as a reference protein database and trypsin was set as a digestion enzyme, while Carbamidomethylation on Cys residues was set as static modification and Oxidation on Met as dynamic modification. The lowest charge state was set as 2, while the highest as 6; match tolerance on spectra was set at 0.02 Da. Precursor Mass Tolerance was set at 10 ppm, and Fragment Mass Tolerance at 0.02 Da. The FDR threshold was set at 0.01 (strict). A single Processing step was performed for each produced spectrum file (24 raw spectra files in total), and subsequently, a single Consensus step with the 24 MSF Files resulting from Processing was performed with the following nodes: (1) MSF Files; (2) PSM Grouper; (3) Peptide Validator; (4) Peptide and Protein Filter; (5) Protein Annotation; (6) Protein Scorer; (7) Protein FDR Validator; and (8) Protein Grouping. The resulting protein matrix, containing the Peptide Spectral Matches (PSMs, or SpCs, Spectral Counts) of every identified protein in each single sample was exported in .xlsx format for further elaboration.

A protein–protein interaction (PPI) network model was reconstructed by STRING Cytoscape’s APP [51] (version 2.2.0, released 30 December 2024) starting from a total of 677 DEPs selected by LDA (*p* < 0.05). Only protein–protein interactions “databases” and/or “experiments” annotated with a Score ≥ 0.3 and ≥ 0.15 were retained; a network of 663 nodes and 5065 interactions was built. The reconstructed network was grouped in PPI functional modules by the support of STRING Cytoscape’s APP and BINGO 3.05 [52]; as for BINGO 2.44, Homo sapiens organism, a hypergeometric test and Benjamini–Hochberg FDR correction (≤0.01) were set.

### 4.11. Statistics

Statistical analysis was carried out using Prism 8.4.2 software (GraphPad, La Jolla, CA, USA). Data were expressed as mean ± SEM. A statistically significant number of repetitions were performed for each measurement, usually three unless otherwise stated. Differences among groups were calculated as one-way and two-way ANOVAs with multiple comparisons testing (Tukey). A *p*-value < 0.05 was considered statistically significant. Statistically significant values are presented as * *p* < 0.05, ** *p* < 0.01, *** *p* < 0.001, and **** *p* < 0.0001.

## 5. Conclusions

In conclusion, this study establishes a proof-of-concept for CSPG4.CAR-T therapy as a novel and targeted approach for treating DMD-associated cardiomyopathy. By reducing fibrosis, promoting neuroimmune remodeling, and enhancing cardiac function, CSPG4.CAR-T cells address multiple pathological components of the dystrophic heart. These findings highlight the potential of ECM-targeted immunotherapies to modulate disease progression in non-oncologic settings.

Future research should focus on optimizing CAR design, evaluating long-term safety and efficacy, and defining the mechanisms underlying cardiac benefit. In particular, combinatorial CAR-T strategies, transient CAR expression systems, or earlier therapeutic interventions may enhance clinical applicability and safety. Parallel studies exploring the impact of CSPG4-targeted therapy on other DMD-affected tissues, such as skeletal muscle, could further broaden its therapeutic value. Given the urgent unmet need for disease-modifying treatments in DMD cardiomyopathy, CSPG4-targeted CAR-T therapy represents a promising step toward precision medicine approaches for this devastating disorder.

*Competency in medical knowledge*: DMD-associated cardiomyopathy remains a poorly understood and undertreated aspect of the disease, despite being a leading cause of morbidity and mortality. Current therapies fail to directly address the underlying fibrotic and inflammatory processes driving cardiac decline. This study serves as proof-of-concept highlighting CSPG4 as a key contributor to maladaptive ECM remodeling and impaired cardiac innervation. We demonstrated that CSPG4.CAR-T therapy ameliorates fibrosis, restores innervation, and improves cardiac function in a dystrophic mouse model.

*Translational outlook*: This study establishes CSPG4-targeted CAR-T therapy as a multifaceted intervention capable of remodeling the dystrophic cardiac microenvironment. Beyond antifibrotic effects, this approach supports neurovascular reintegration and myocardial repair, indicating that ECM modulation may indirectly foster structural and functional recovery. The favorable tolerability and immunomodulatory effects observed in *mdx* mice suggest that CSPG4.CAR-T therapy may be a viable and scalable strategy for DMD patients and potentially other dystrophy-related cardiomyopathies.

## Figures and Tables

**Table 1:** List of mouse primers.

Gene Symbol	Sense-Forward Primer	Antisense-Reverse Primer
*Col1a1*	CCTCAGGGTATTGCTGGACA	GAAGGACCTTGTTTGCCAGG
*Col3a1*	CCCAACCCAGAGATCCCATT	GGTCACCATTTCTCCCAGGA
*Fibronectin*	CATGCCTCGGGAATGGAAAG	TGCTTCATGGGGATCACACT
*Tgf-β1*	CAACCCAGGTCCTTCCTAAA	GGAGAGCCCTGGATACCAAC
*Adgre1*	CGTGTTGTTGGTGGCACTGTGA	CCACATCAGTGTTCCAGGAGAC
*Cspg4*	AAGGAAGTGCAGAGGAGGTC	TGAGGACAGTAGGAGACCGA
*Th*	CAATACAAGCAGGGTGAGCC	TAGCATAGAGGCCCTTCAGC
*Ngf*	ATACTGCACCACGACTCACA	GGCTGTGTCTATCCGGATGA
*Il-1α*	ACGGCTGAGTTTCAGTGAGACC	CACTCTGGTAGGTGTAAGGTGC
*Il-1β*	TGGACCTTCCAGGATGAGGACA	GTTCATCTCGGAGCCTGTAGTG
*Tubb3*	AACCTGGAACCATGGACAGT	AGCACCACTCTGACCAAAGA
*Eef1a2*	CAAGATTGGGGGCATTGGGA	TCGCTAAGTGCCTCATGGTG

**Table 2:** Exploris 240 mass spectrometer method summary.

Exploris 240 Parameter	
**Full scan**	
Resolution MS1	120,000
Scan range lower limit, *m*/*z*	375
Scan range upper limit, *m*/*z*	1200
AGC target custom, normalized %	300
Maximum injection time mode	auto
RF lens %	80
**Filters**	
**MIPS**	
Monoisotopic peak det.	peptide
**Intensity**	
Intensity threshold	8.0 × 10^3^
**Charge state**	
Include charge state	2–6
**Dynamic Exclusion custom**	
Exclude after n times	1
Exclusion duration (s)	15
Mass tolerance ppm	10
**ddMS^2^ (top 20 scans)**	
Resolution MS2	15,000
AGC target custom, normalized %	100
Maximum injection time, ms	auto
Scan range lower limit, *m*/*z*	120
Scan range upper limit, *m*/*z*	1200
Isolation window, *m*/*z*	2
HCD collision energy, %	30

## Data Availability

The data that support the findings of this study are available from the corresponding author upon request. Raw mass spectra related to proteomic analysis are publicly available in the MassIVe database under the ID MSV000097631, or by using the link ftp://MSV000097631@massive-ftp.ucsd.edu (accessed on 7 July 2025).

## Supplementary Materials

The following supporting information can be downloaded at: https://www.mdpi.com/article/10.3390/ijms26146590/s1.

## Abbreviations

The following abbreviations are used in this manuscript:

CAR	Chimeric Antigen Receptor
CSPG4	Chondroitin Sulfate Proteoglycan 4
cTNNT	Cardiac Troponin T
DMD	Duchenne Muscular Dystrophy
ECM	Extracellular Matrix
EF	Ejection Fraction
FS	Fractional Shortening
NCJ	Neurocardiac Junction
SYN1	Synapsin 1
TH	Tyrosine Hydroxylase
