# Supplementary material for: CSPG4.CAR-T Cells Modulate Extracellular Matrix Remodeling in DMD Cardiomyopathy
Source: Int J Mol Sci. 2025 Jul 9;26(14):6590. doi: 10.3390/ijms26146590 (PMC12294788; doi:10.3390/ijms26146590)
Supplement: Supplementary file 1 [file ijms-26-06590-s001.zip › ijms-3722425_Supplemental Material_Figure_S1.pdf]

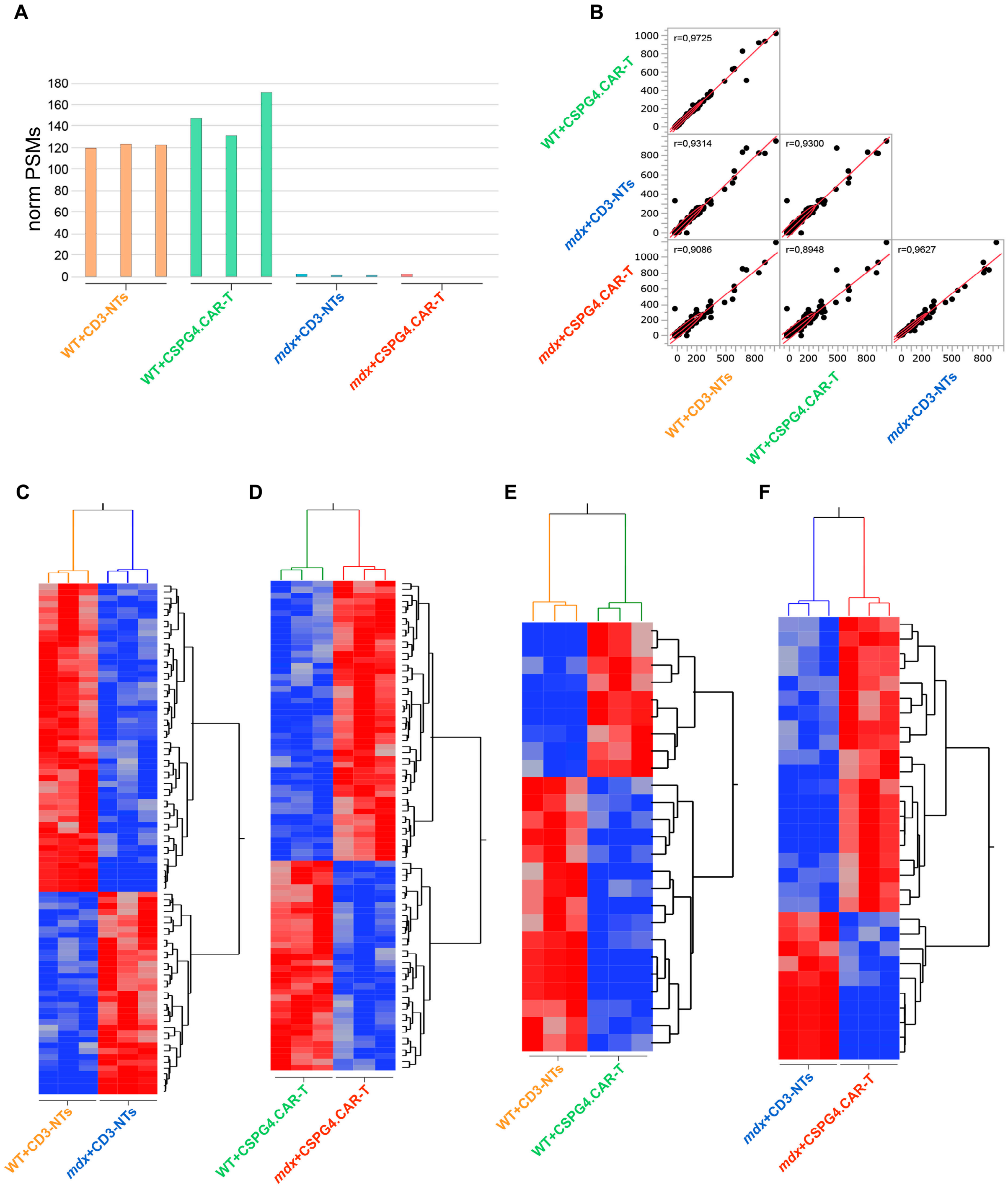

Figure S1. Differentially expressed proteins (DEPs) per pairwise comparison. A) Expression of Dmd protein in WT+CD3-NTs, WT+CSPG4.CAR-T, *mdx*+CD3-NTs and *mdx*+CSPG4.CAR-T; protein relative abundance is expressed in norm PSMs, and three different biological replicates *per* condition are shown**. B)** Multivariate correlation analysis (Spearman's correlation) using all DEPs selected by LDA (P<0.01) in all considered pairwise comparisons. **C)** Hierarchical Clustering (HC) using DEPs (P<0.01) from WT+CD3-NTs vs *mdx*+CD3-NTs, **D)** WT+CSPG4.CAR-T vs *mdx*+CSPG4.CAR-T, **E)** WT+CD3-NTs vs WT+CSPG4.CAR-T and **F)** *mdx*+CD3-NTs vs *mdx*+CSPG4.CAR-T.
